# Supplementary figures and images for: Antagonistic control of DDK binding to licensed replication origins by Mcm2 and Rad53
Source: eLife. 2020 Jul 23;9:e58571. doi: 10.7554/eLife.58571 (PMC7398698; doi:10.7554/eLife.58571)

Figure 1- source data 1

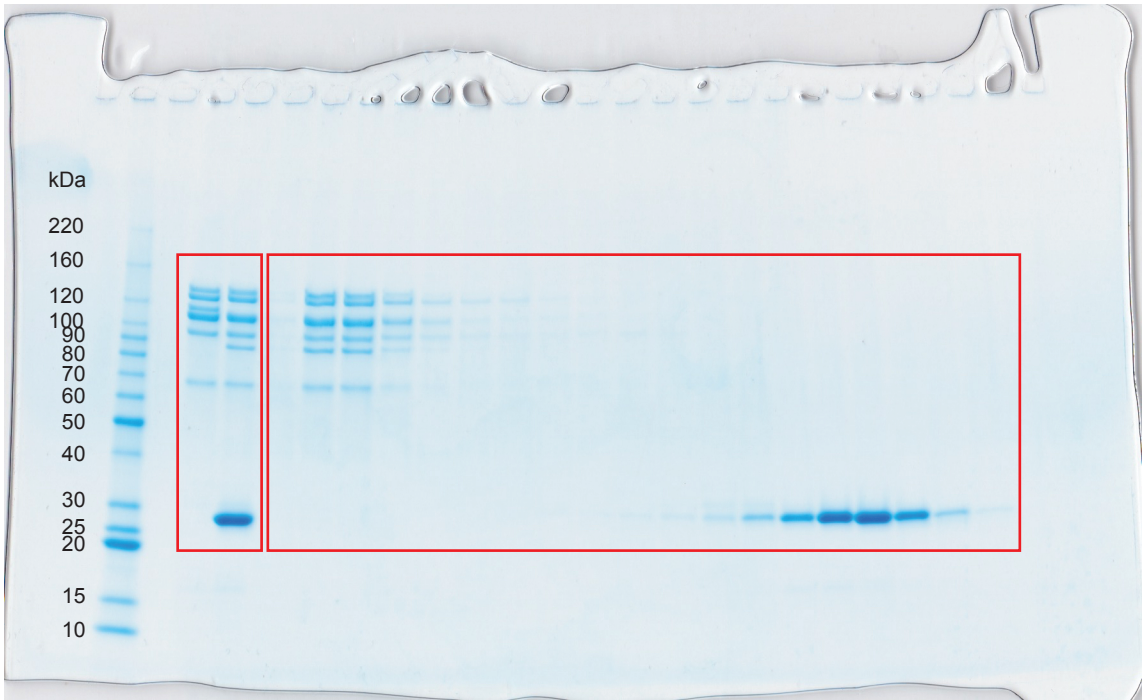

Figure 1B + D

Supplement: Figure 1—source data 1. [file elife-58571-fig1-data1.pdf]

Figure 1- source data 2

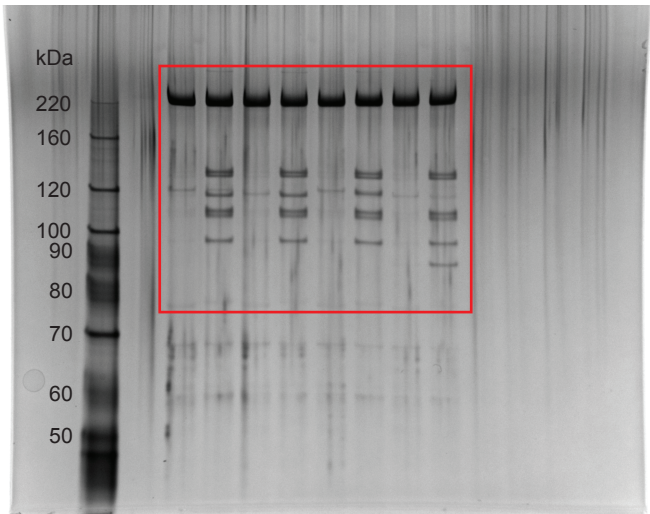

Figure 1C

Supplement: Figure 1—source data 2. [file elife-58571-fig1-data2.pdf]

**Figure 1- source data 3**

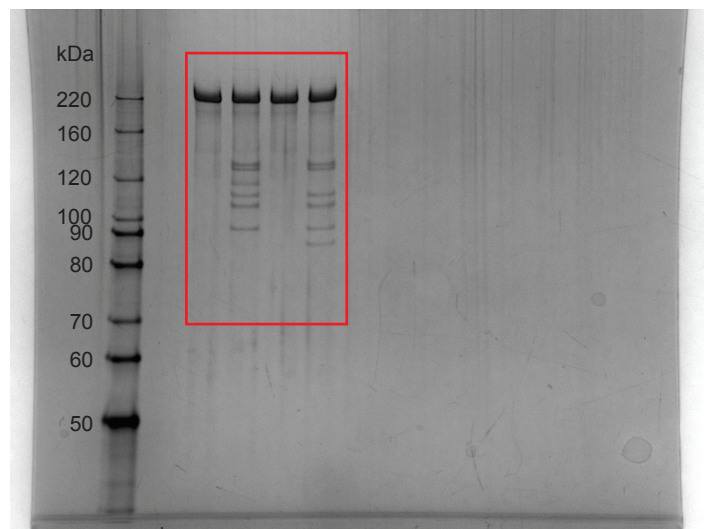

**Figure 1 Panel E**

Supplement: Figure 1—source data 3. [file elife-58571-fig1-data3.pdf]

## Figure 1 - figure supplement 1 - source data 1

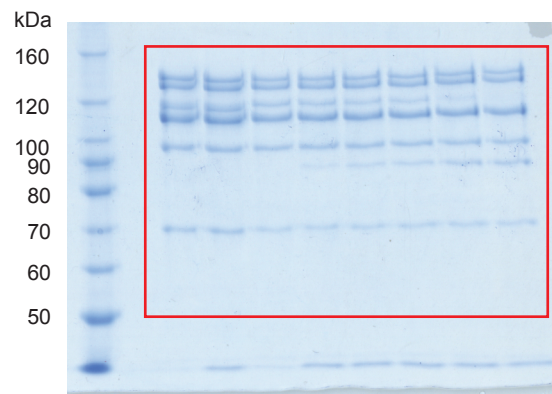

Figure 1 - figure supplement 1

Supplement: Figure 1—figure supplement 1—source data 1. [file elife-58571-fig1-figsupp1-data1.pdf]

Figure 2 - source data 1

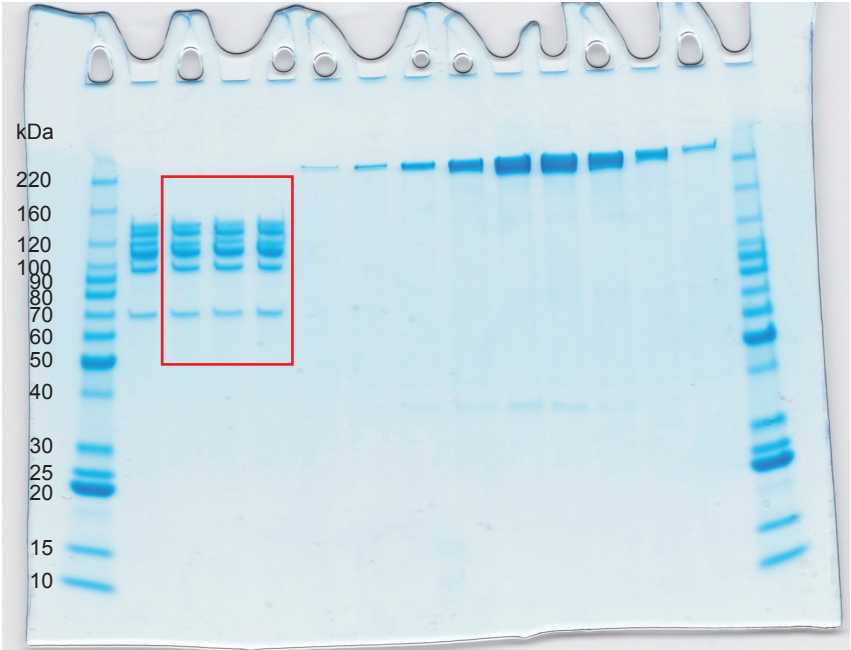

Figure 2A

Supplement: Figure 2—source data 1. [file elife-58571-fig2-data1.pdf]

**Figure 2 - source data 2**

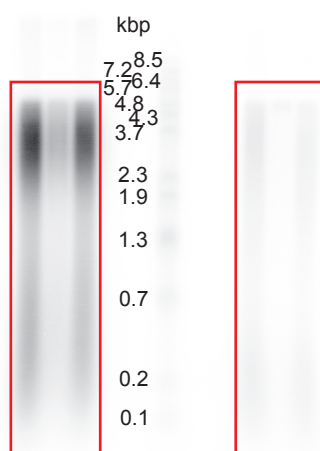

**Figure 2C**

Supplement: Figure 2—source data 2. [file elife-58571-fig2-data2.pdf]

Figure 2 - figure supplement 1 - source data 1

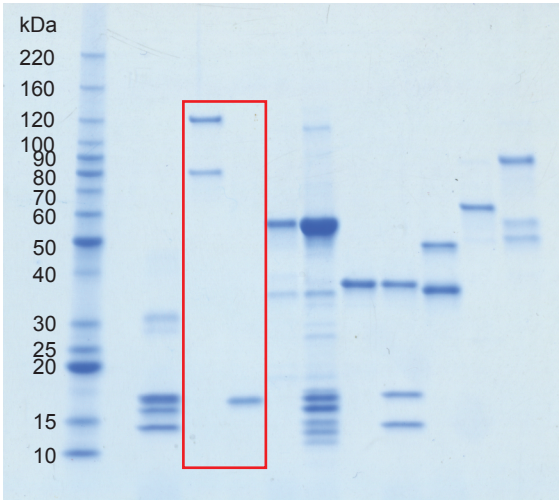

Figure 2 - figure supplement 1A

Supplement: Figure 2—figure supplement 1—source data 1. [file elife-58571-fig2-figsupp1-data1.pdf]

## Figure 2 - figure supplement 1 - source data 2

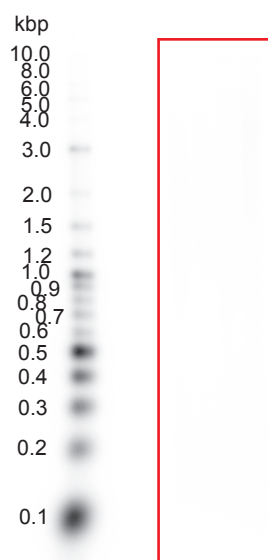

Figure 2 - figure supplement 1B

Supplement: Figure 2—figure supplement 1—source data 2. [file elife-58571-fig2-figsupp1-data2.pdf]

Figure 2 - figure supplement 2 - source data 1

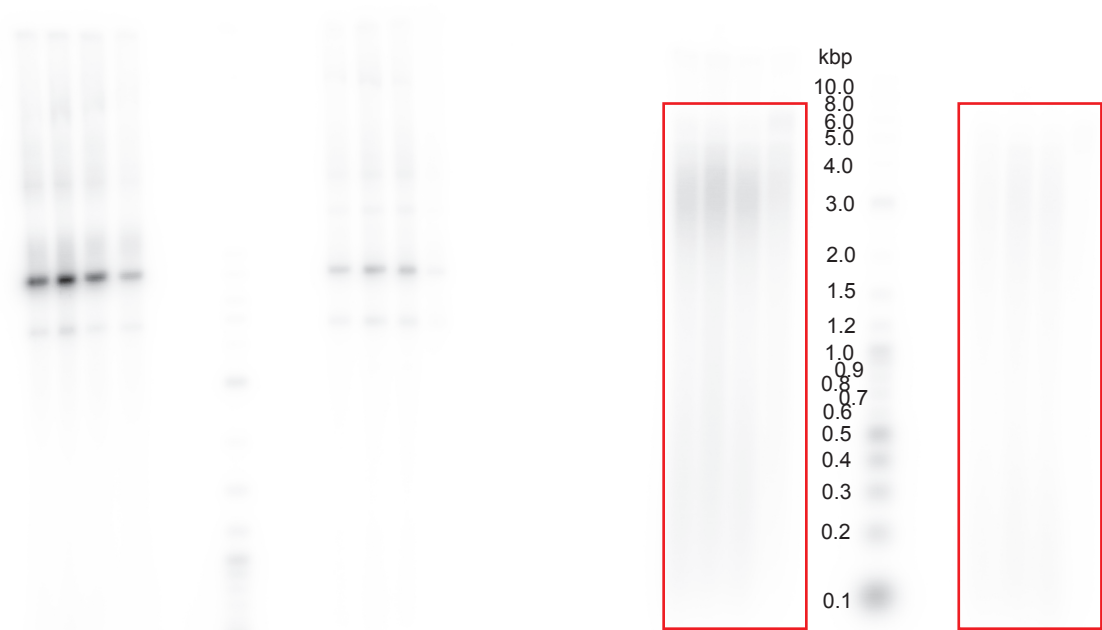

Figure 2 - figure supplement 2

Supplement: Figure 2—figure supplement 2—source data 1. [file elife-58571-fig2-figsupp2-data1.pdf]

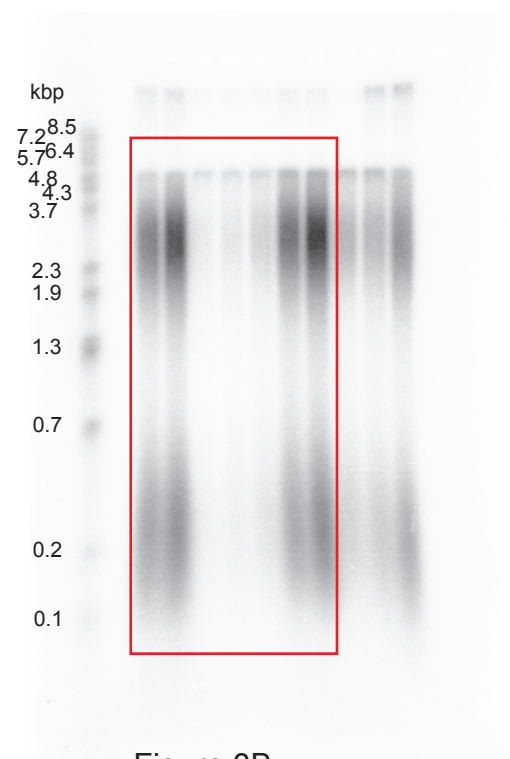

Figure 3B  
Autoradiograph

Supplement: Figure 3—source data 1. [file elife-58571-fig3-data1.pdf]

Figure 3 - source data 2

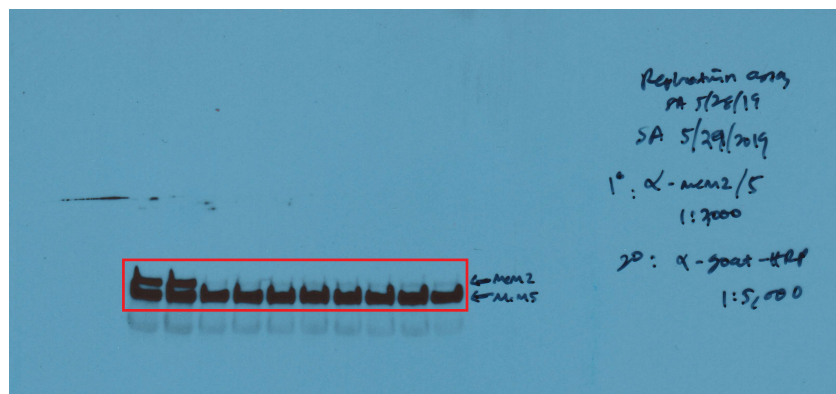

Figure 3B  
Immunoblot: Mcm2, Mcm5

Supplement: Figure 3—source data 2. [file elife-58571-fig3-data2.pdf]

Figure 3 - source data 3

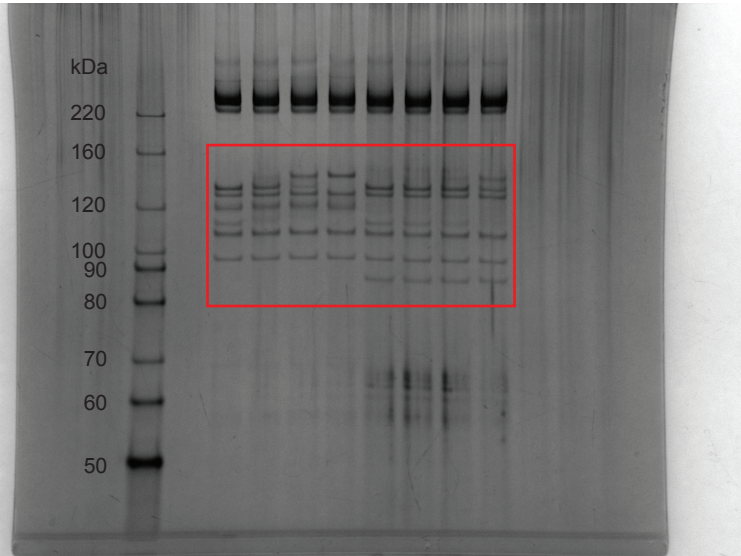

Figure 3C  
Silver stain

Supplement: Figure 3—source data 3. [file elife-58571-fig3-data3.pdf]

## Figure 3 - source data 4

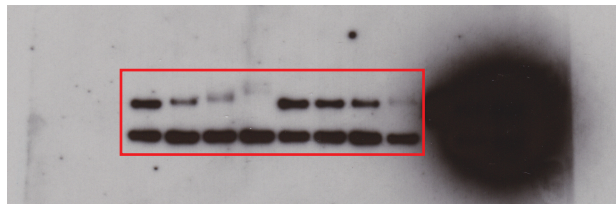

Figure 3C  
Immunoblot: Mcm4, Mcm5

Supplement: Figure 3—source data 4. [file elife-58571-fig3-data4.pdf]

**Figure 3 - source data 5**

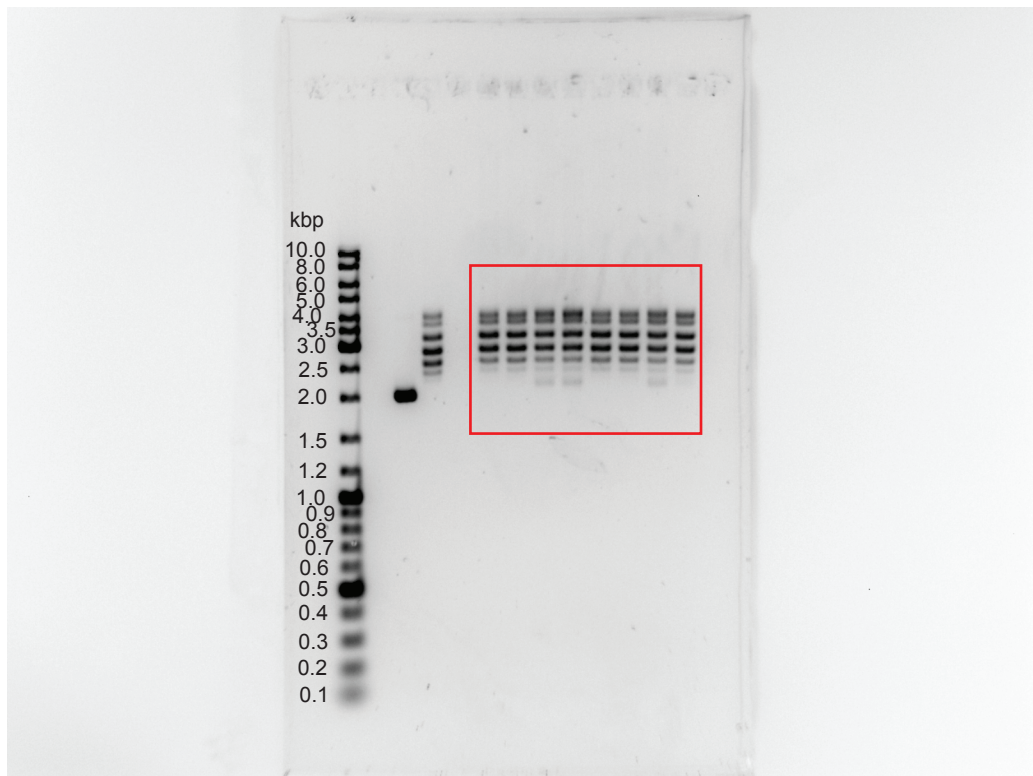

**Figure 3D**

Supplement: Figure 3—source data 5. [file elife-58571-fig3-data5.pdf]

Figure 4 - source data 1

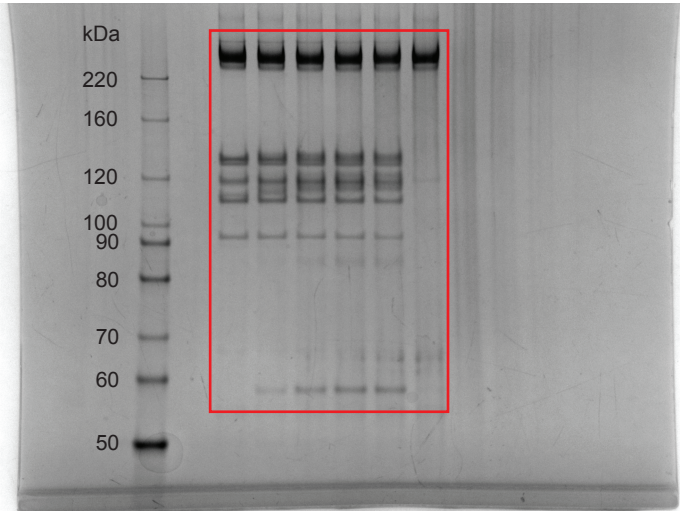

Figure 4A  
Silver stain

Supplement: Figure 4—source data 1. [file elife-58571-fig4-data1.pdf]

Figure 4 - source data 2

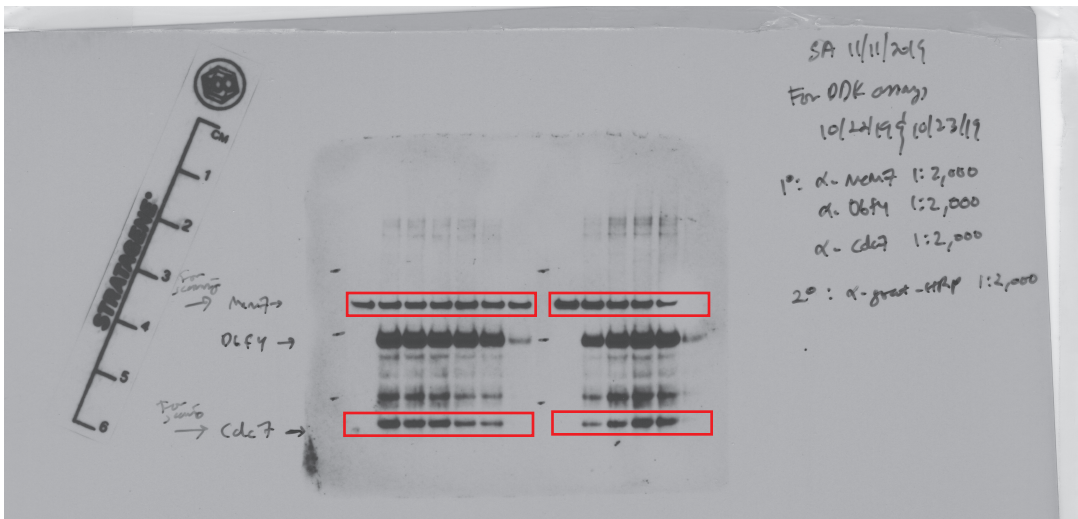

Figure 4A+E  
Immunoblot: Mcm7, Cdc7

Supplement: Figure 4—source data 2. [file elife-58571-fig4-data2.pdf]

Figure 4 - source data 3

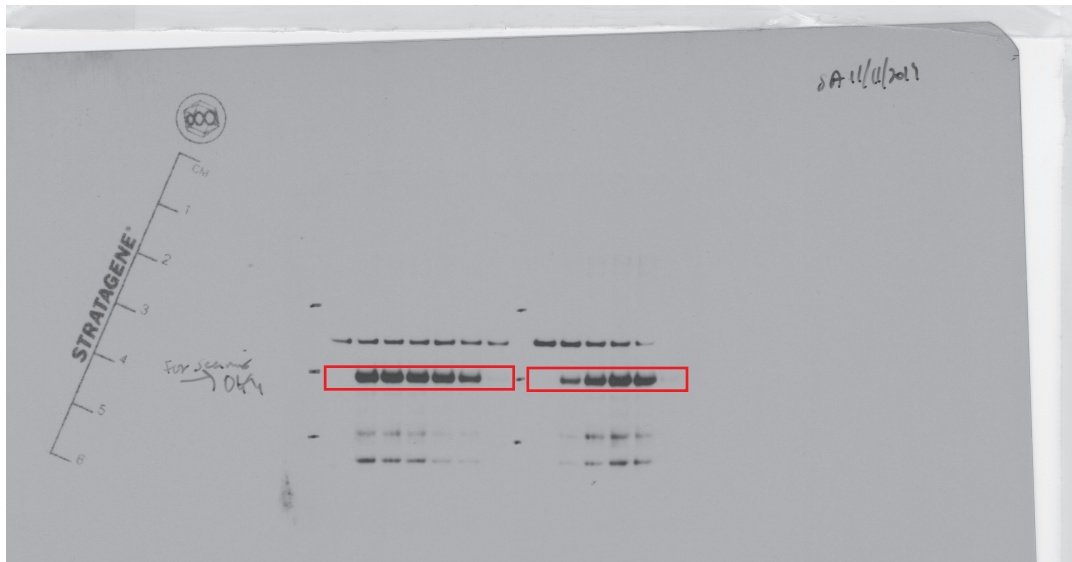

Figure 4A+E  
Immunoblot: Dbf4

Supplement: Figure 4—source data 3. [file elife-58571-fig4-data3.pdf]

**Figure 4 - source data 4**

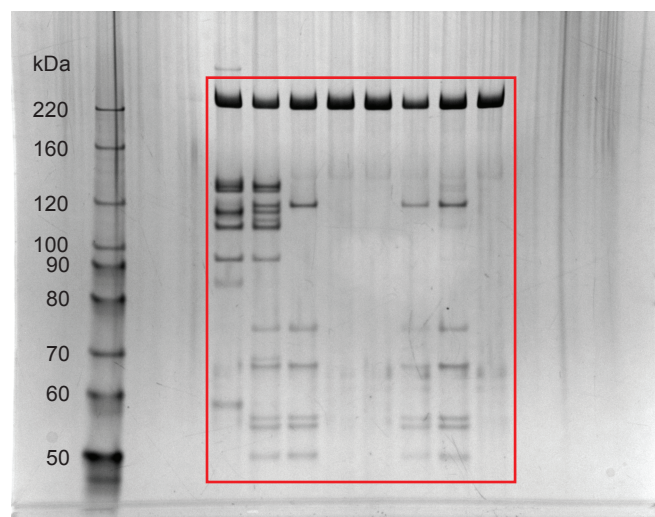

Figure 4B  
Silver stain

Supplement: Figure 4—source data 4. [file elife-58571-fig4-data4.pdf]

Figure 4 - source data 5

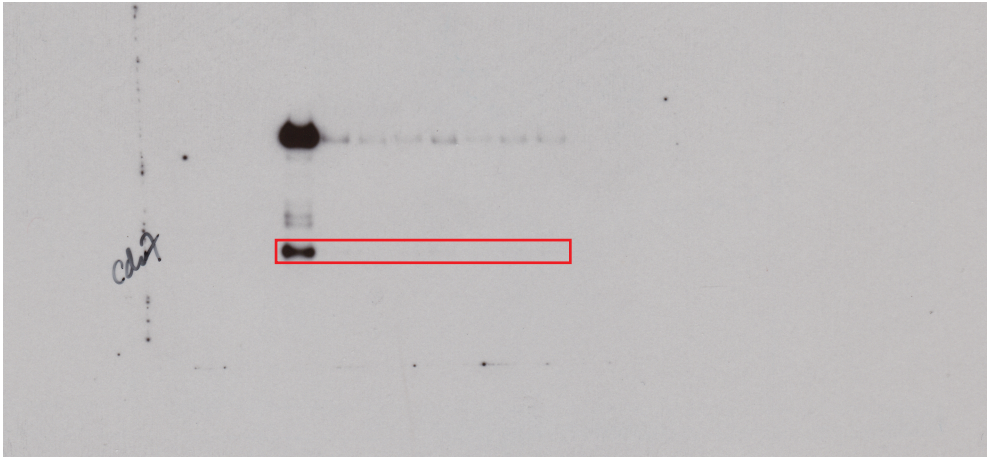

Figure 4B  
Immunoblot: Cdc7

Supplement: Figure 4—source data 5. [file elife-58571-fig4-data5.pdf]

## Figure 4 - source data 6

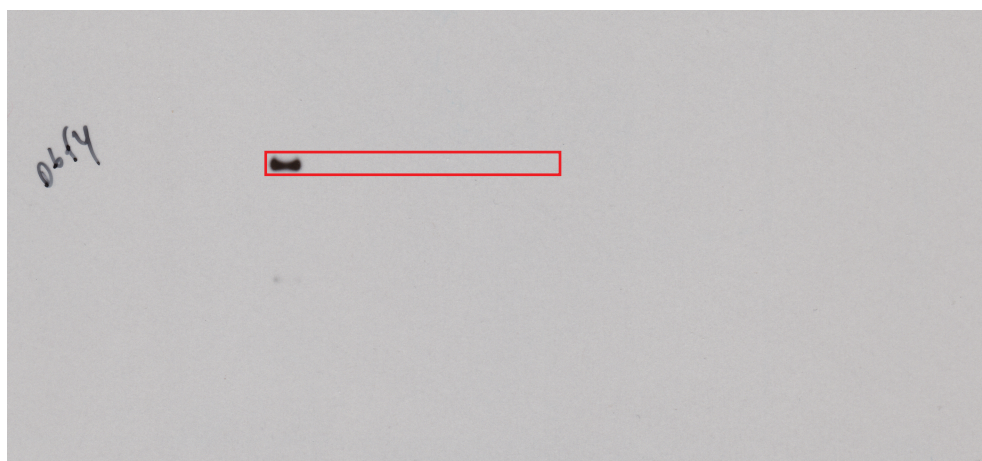

Figure 4B  
Immunoblot: Dbf4

Supplement: Figure 4—source data 6. [file elife-58571-fig4-data6.pdf]

Figure 4 - source data 7

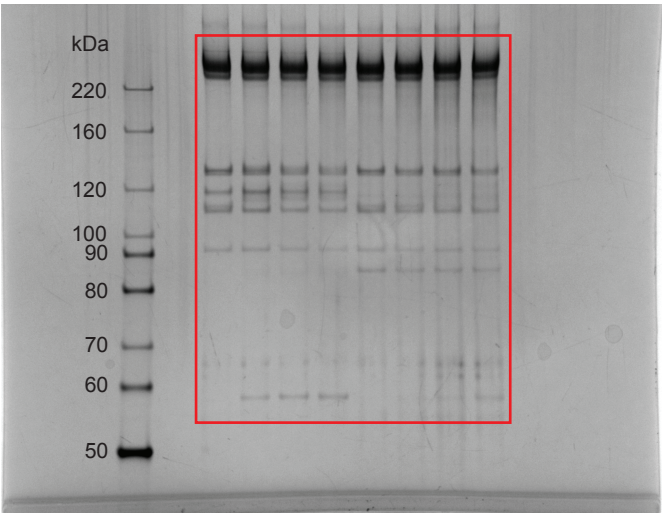

Figure 4C  
Silver stain

Supplement: Figure 4—source data 7. [file elife-58571-fig4-data7.pdf]

Figure 4 - source data 8

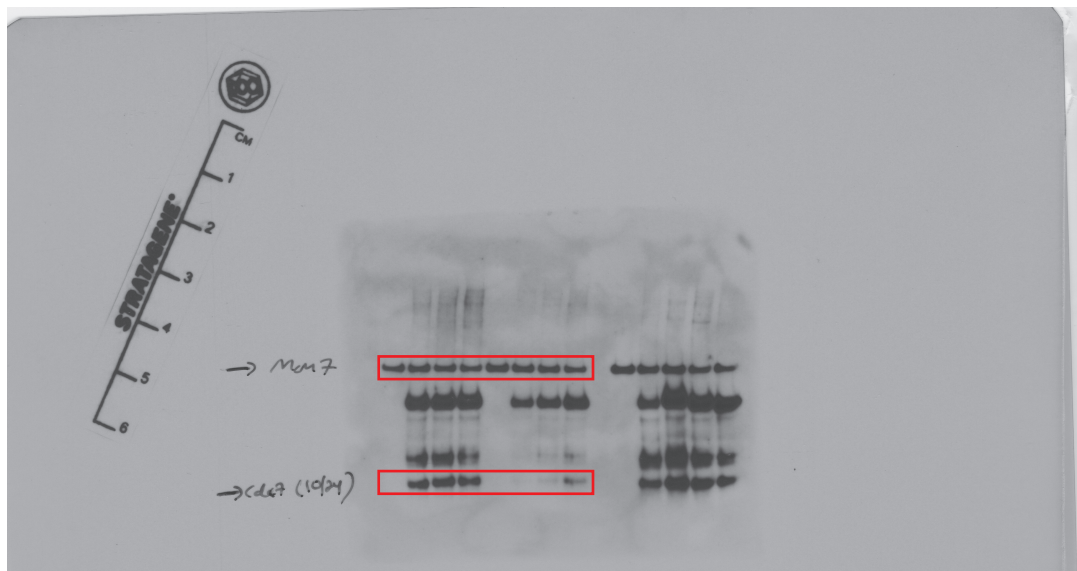

Figure 4C  
Immunoblot: Mcm7, Cdc7

Supplement: Figure 4—source data 8. [file elife-58571-fig4-data8.pdf]

Figure 4 - source data 9

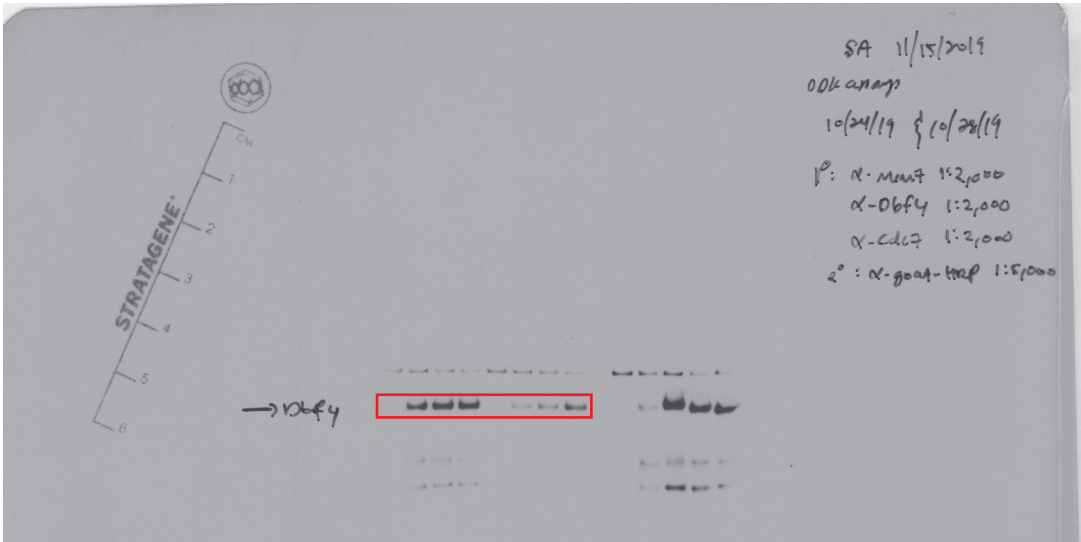

Figure 4C  
Immunoblot: Dbf4

Supplement: Figure 4—source data 9. [file elife-58571-fig4-data9.pdf]

Figure 4 - source data 10

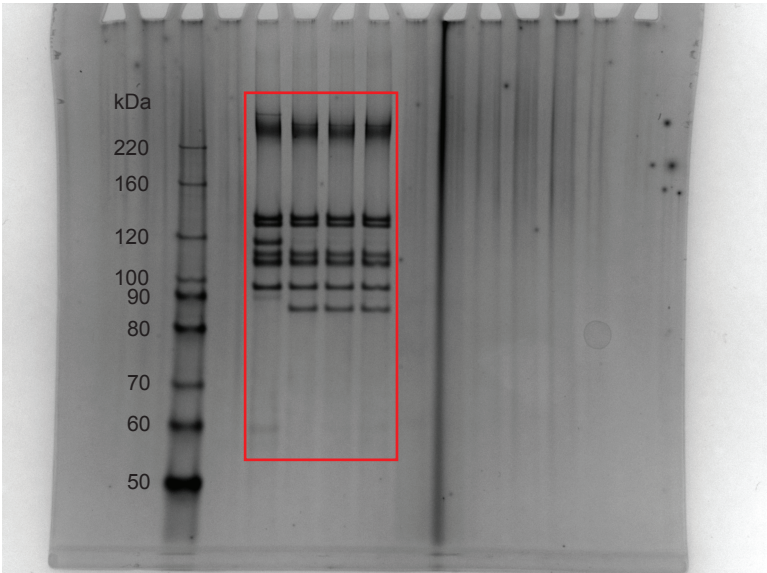

Figure 4D  
Silver stain

Supplement: Figure 4—source data 10. [file elife-58571-fig4-data10.pdf]

## Figure 4 - source data 11

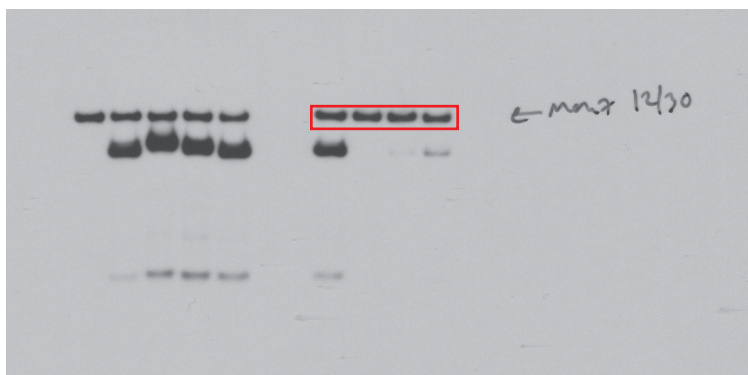

Figure 4D  
Immunoblot: Mcm7

Supplement: Figure 4—source data 11. [file elife-58571-fig4-data11.pdf]

Figure 4 - source data 12

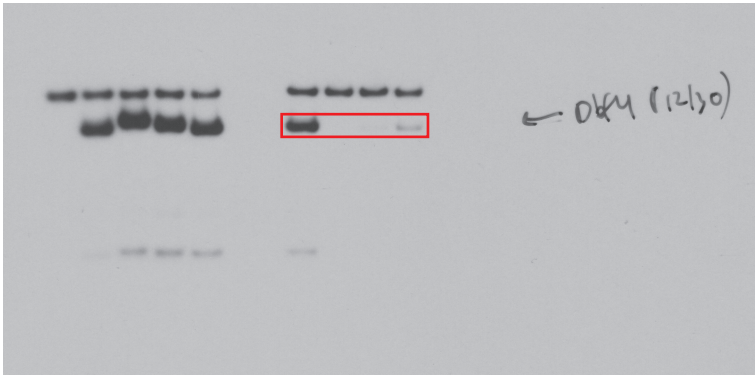

Figure 4D  
Immunoblot: Dbf4

Supplement: Figure 4—source data 12. [file elife-58571-fig4-data12.pdf]

## Figure 4 - source data 13

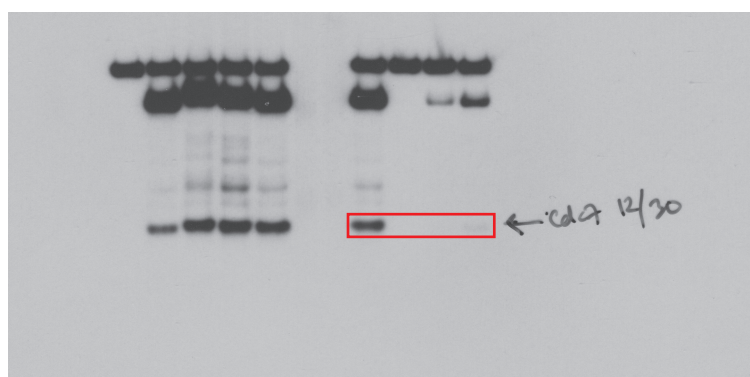

Figure 4D  
Immunoblot: Cdc7

Supplement: Figure 4—source data 13. [file elife-58571-fig4-data13.pdf]

**Figure 4 - source data 14**

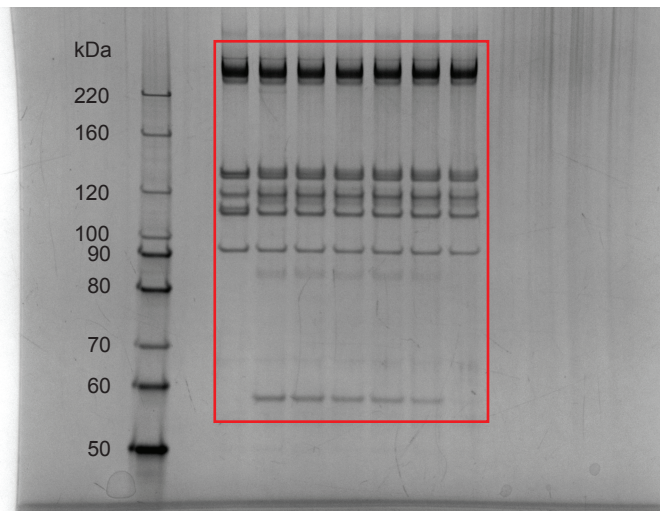

Figure 4E  
Silver stain

Supplement: Figure 4—source data 14. [file elife-58571-fig4-data14.pdf]

## Figure 4 - source data 15

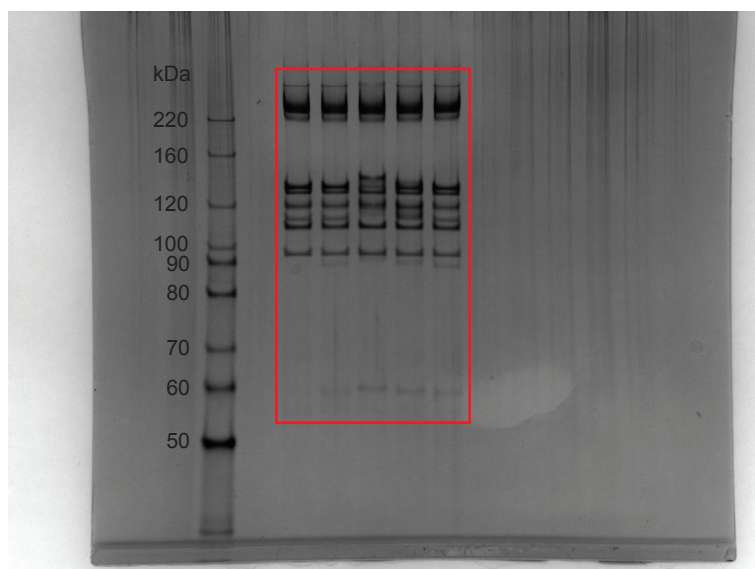

Figure 4F  
Silver stain

Supplement: Figure 4—source data 15. [file elife-58571-fig4-data15.pdf]

Figure 4 - source data 16

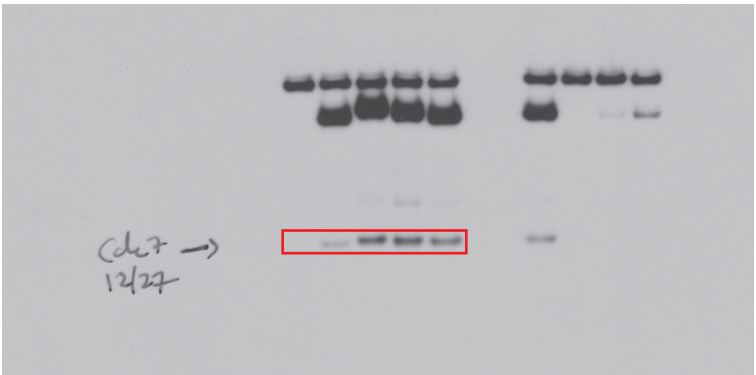

Figure 4F  
Immunoblot: Cdc7

Supplement: Figure 4—source data 16. [file elife-58571-fig4-data16.pdf]

Figure 4 - source data 17

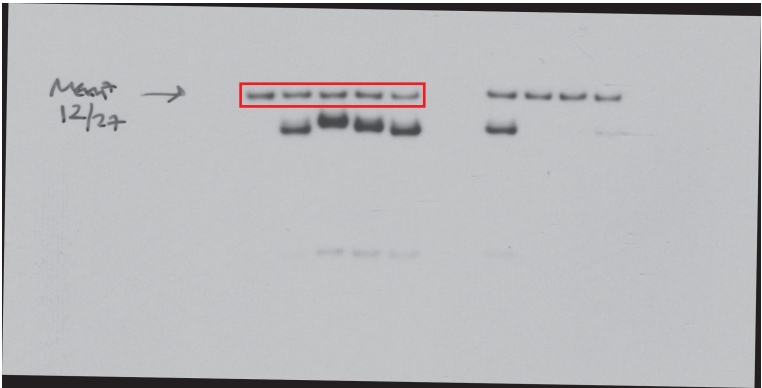

Figure 4F  
Immunoblot: Mcm7

Supplement: Figure 4—source data 17. [file elife-58571-fig4-data17.pdf]

Figure 4 - source data 18

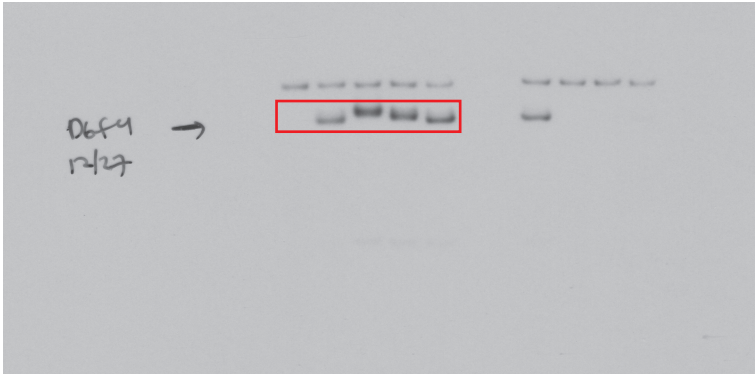

Figure 4F  
Immunoblot: Dbf4

Supplement: Figure 4—source data 18. [file elife-58571-fig4-data18.pdf]

## Figure 5 - source data 1

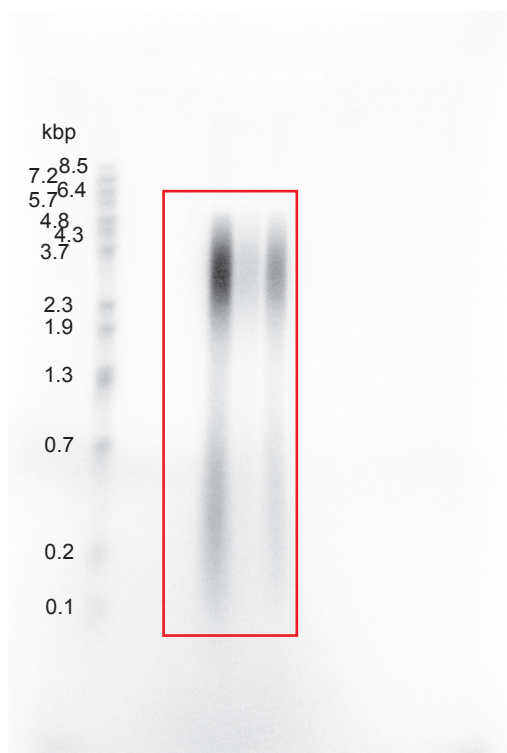

Figure 5A  
Autoradiograph

Supplement: Figure 5—source data 1. [file elife-58571-fig5-data1.pdf]

## Figure 5 - figure supplement 1 - source data 1

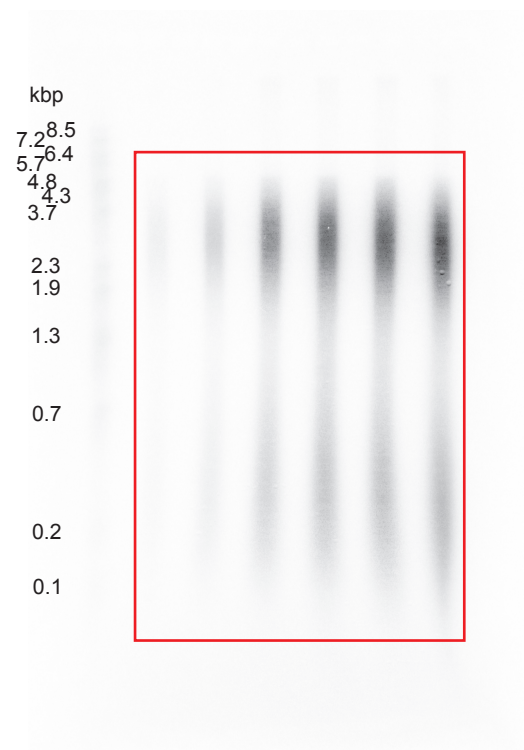

Figure 5 - figure supplement 1A

Supplement: Figure 5—figure supplement 1—source data 1. [file elife-58571-fig5-figsupp1-data1.pdf]

Figure 5 - figure supplement 1 - source data 2

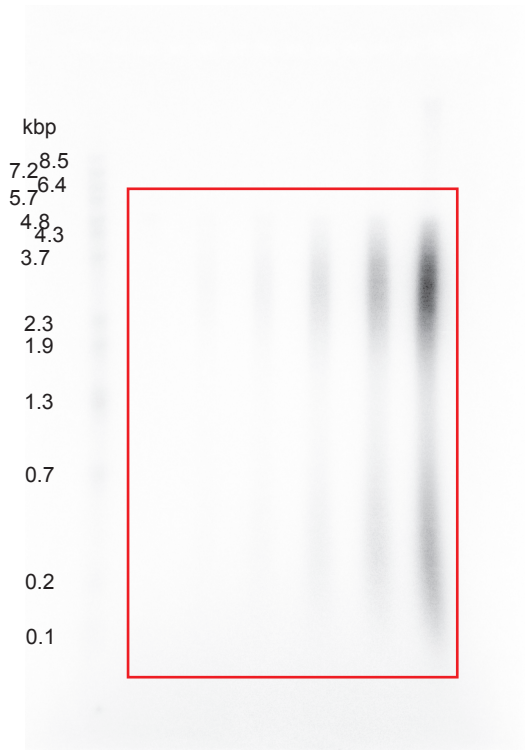

Figure 5 - figure supplement 1B

Supplement: Figure 5—figure supplement 1—source data 2. [file elife-58571-fig5-figsupp1-data2.pdf]

## Figure 6 - source data 1

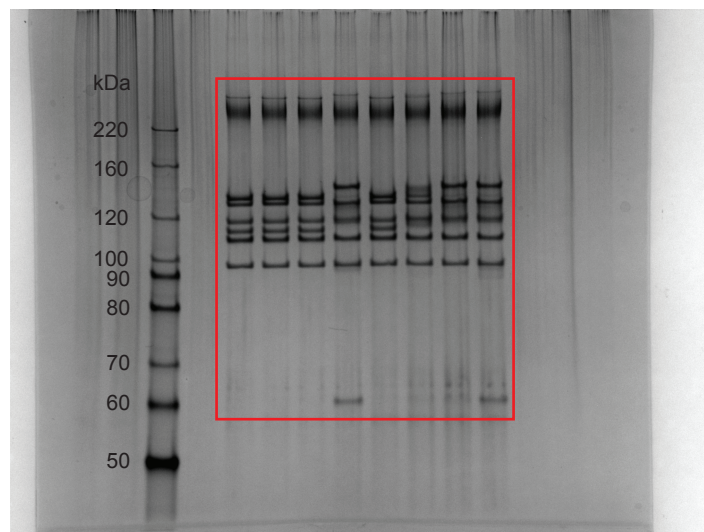

Figure 6A  
Silver stain

Supplement: Figure 6—source data 1. [file elife-58571-fig6-data1.pdf]

## Figure 6 - source data 2

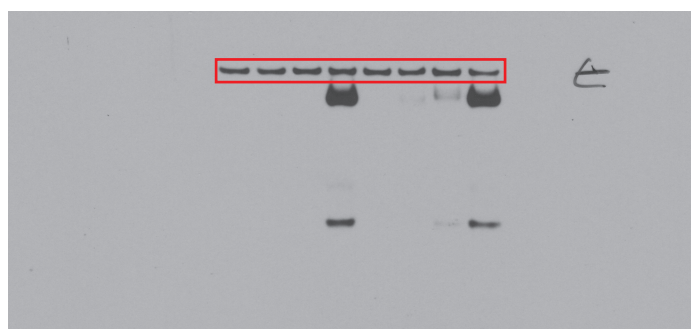

Figure 6A  
Immunoblot Mcm7

Supplement: Figure 6—source data 2. [file elife-58571-fig6-data2.pdf]

## Figure 6 - source data 3

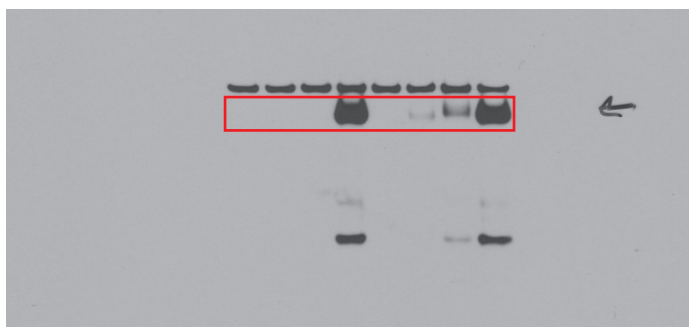

Figure 6A  
Immunoblot: Dbf4

Supplement: Figure 6—source data 3. [file elife-58571-fig6-data3.pdf]

## Figure 6 - source data 4

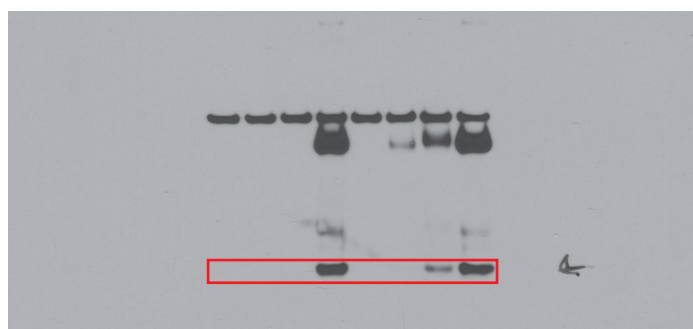

Figure 6A  
Immunoblot: Cdc7

Supplement: Figure 6—source data 4. [file elife-58571-fig6-data4.pdf]

## Figure 6 - source data 5

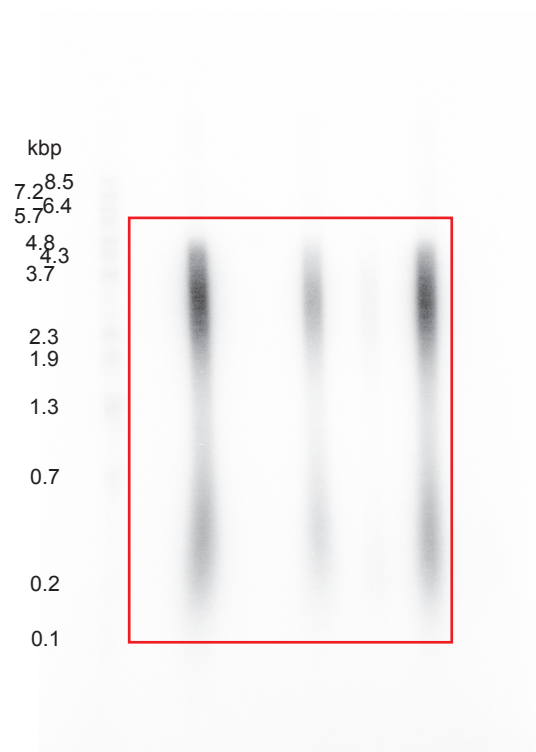

Figure 6B  
Autoradiograph

Supplement: Figure 6—source data 5. [file elife-58571-fig6-data5.pdf]

## Figure 6 - source data 6

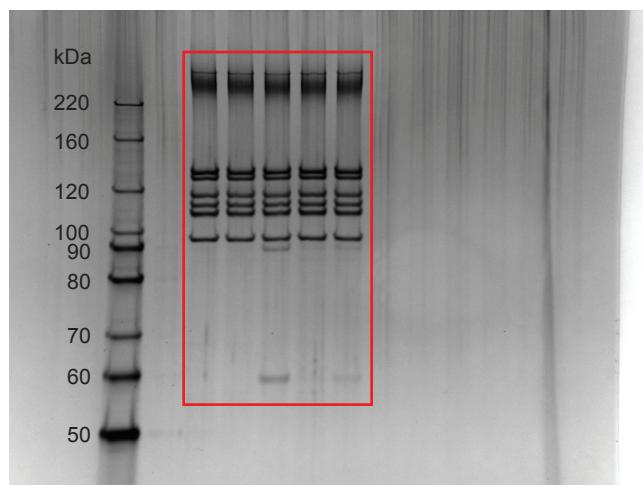

Figure 6C  
Silver stain

Supplement: Figure 6—source data 6. [file elife-58571-fig6-data6.pdf]

Figure 6 - source data 7

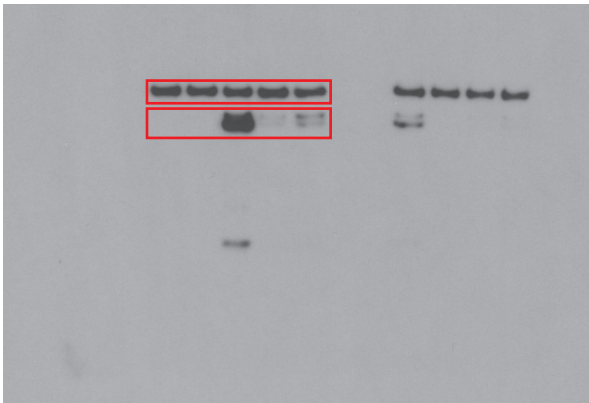

Figure 6C  
Immunoblot:, Mcm7, Dbf4

Supplement: Figure 6—source data 7. [file elife-58571-fig6-data7.pdf]

## Figure 6 - source data 8

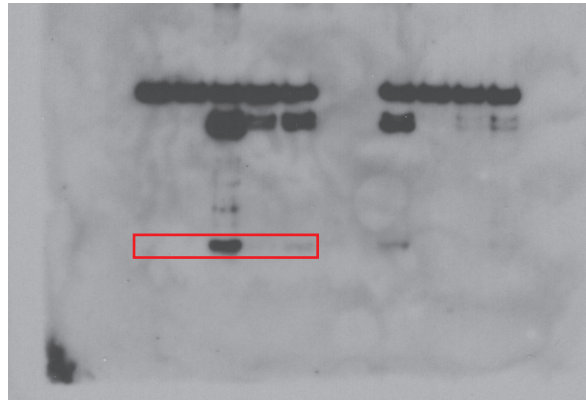

Figure 6C  
Immunoblot: Cdc7

Supplement: Figure 6—source data 8. [file elife-58571-fig6-data8.pdf]

## Figure 7 - source data 1

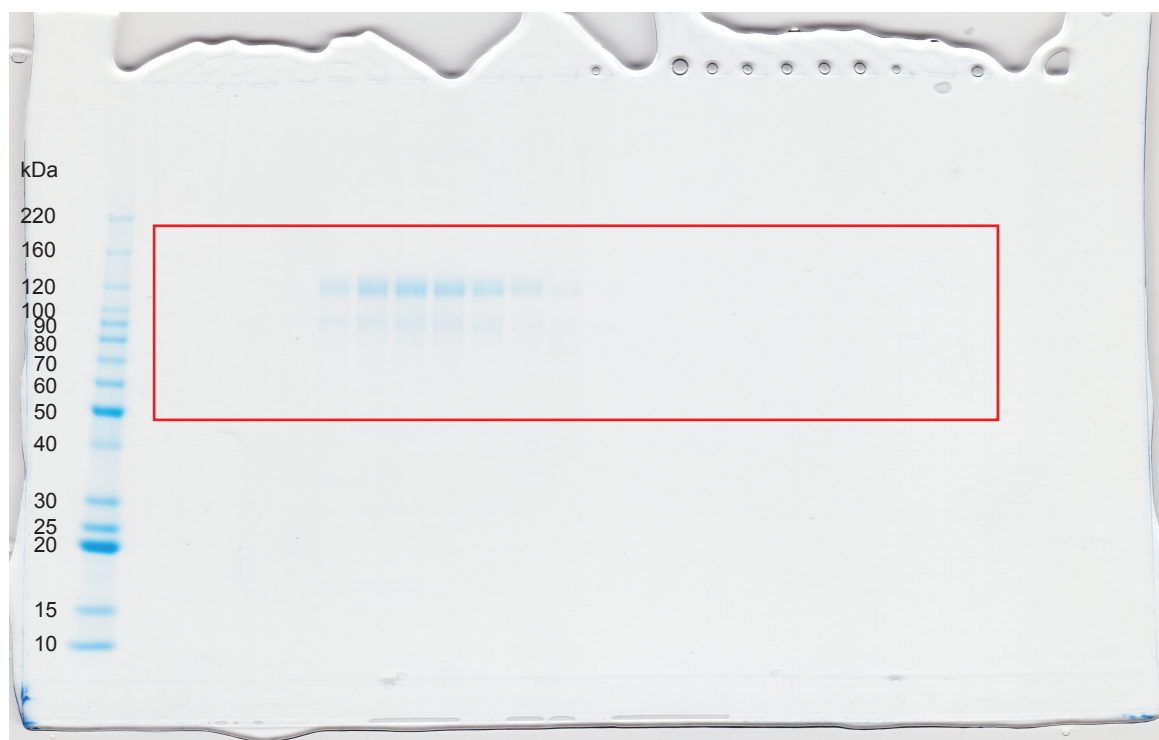

Figure 7A, Rad53-WT

Supplement: Figure 7—source data 1. [file elife-58571-fig7-data1.pdf]

**Figure 7 - source data 2**

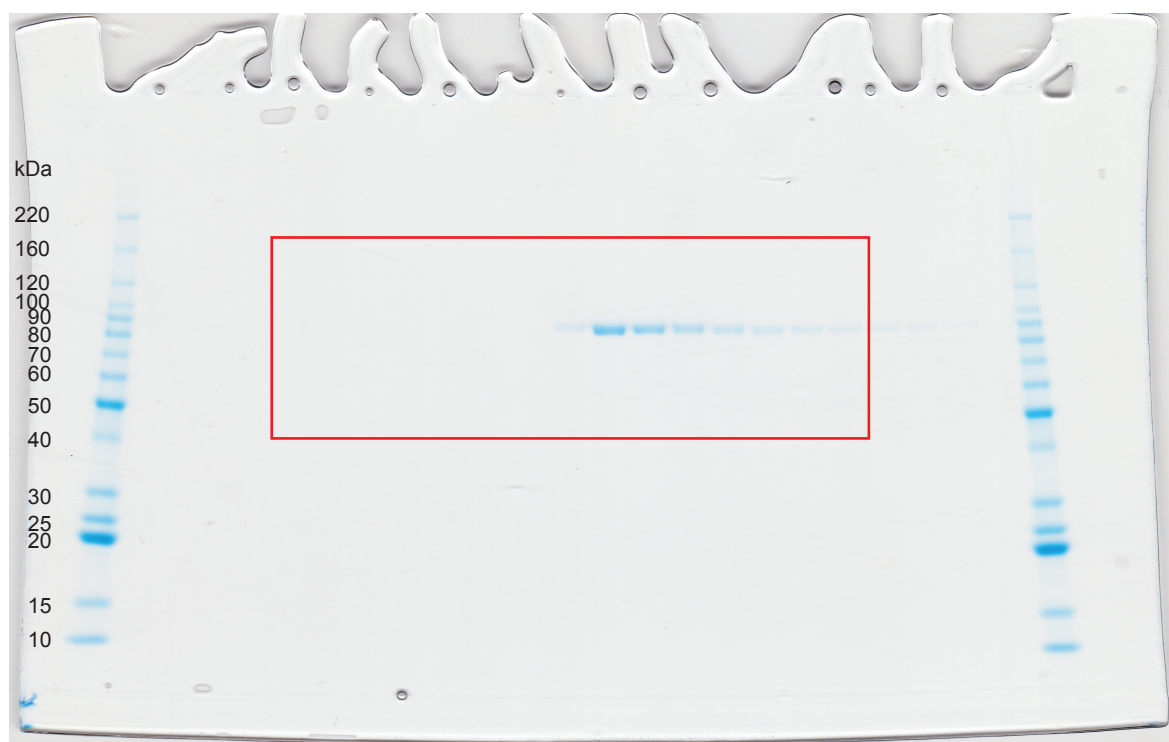

Figure 7A, Rad53-kd

Supplement: Figure 7—source data 2. [file elife-58571-fig7-data2.pdf]

Figure 7 - source data 3

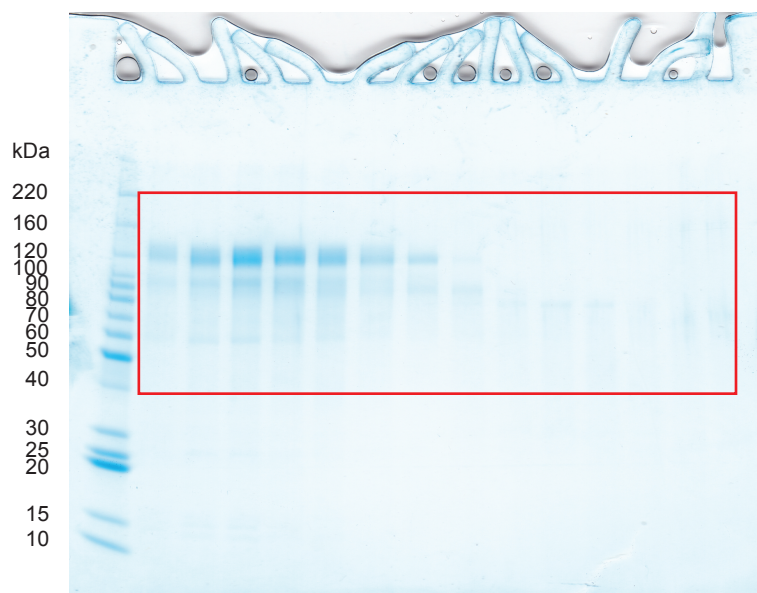

Figure 7B, Rad53-WT + DDK

Supplement: Figure 7—source data 3. [file elife-58571-fig7-data3.pdf]

## Figure 7 - source data 4

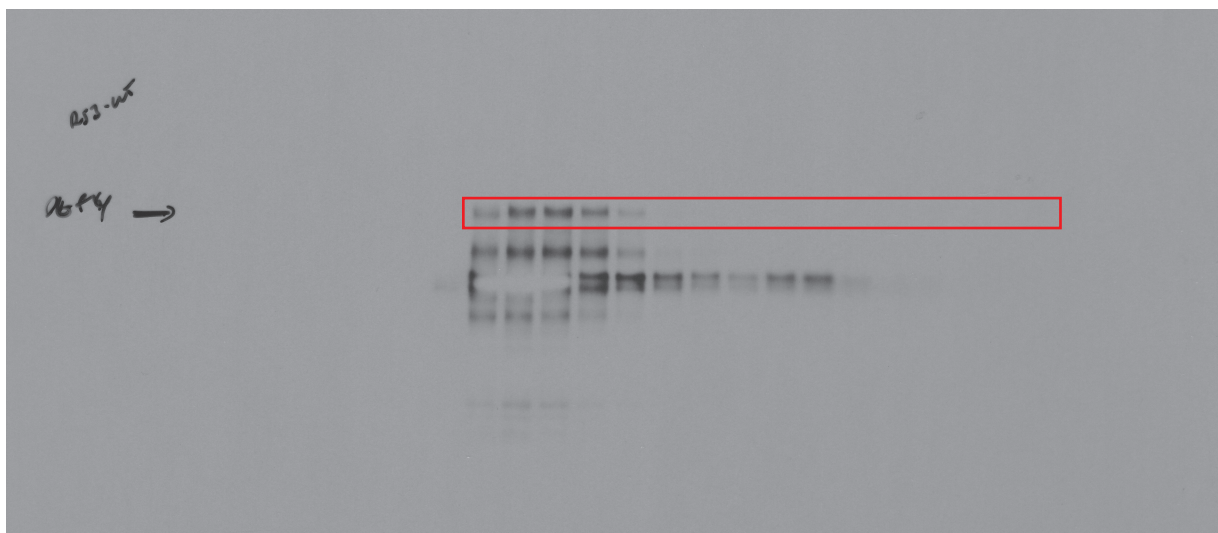

Figure 7B, Rad53-WT + DDK  
Immunoblot: Dbf4

Supplement: Figure 7—source data 4. [file elife-58571-fig7-data4.pdf]

## Figure 7 - source data 5

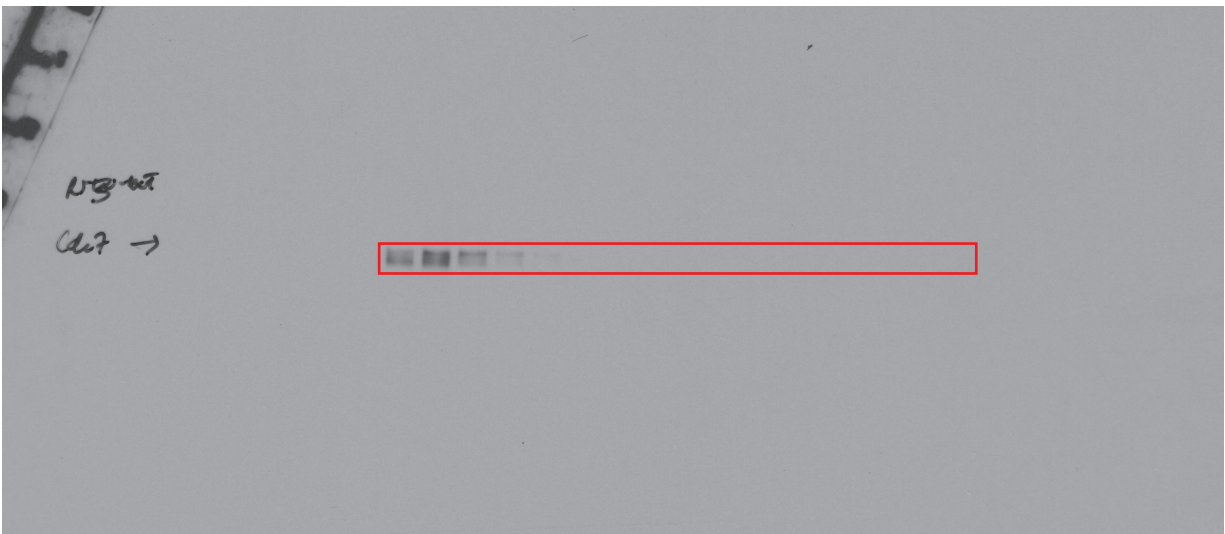

Figure 7B, Rad53-WT + DDK  
Immunoblot: Cdc7

Supplement: Figure 7—source data 5. [file elife-58571-fig7-data5.pdf]

**Figure 7 - source data 6**

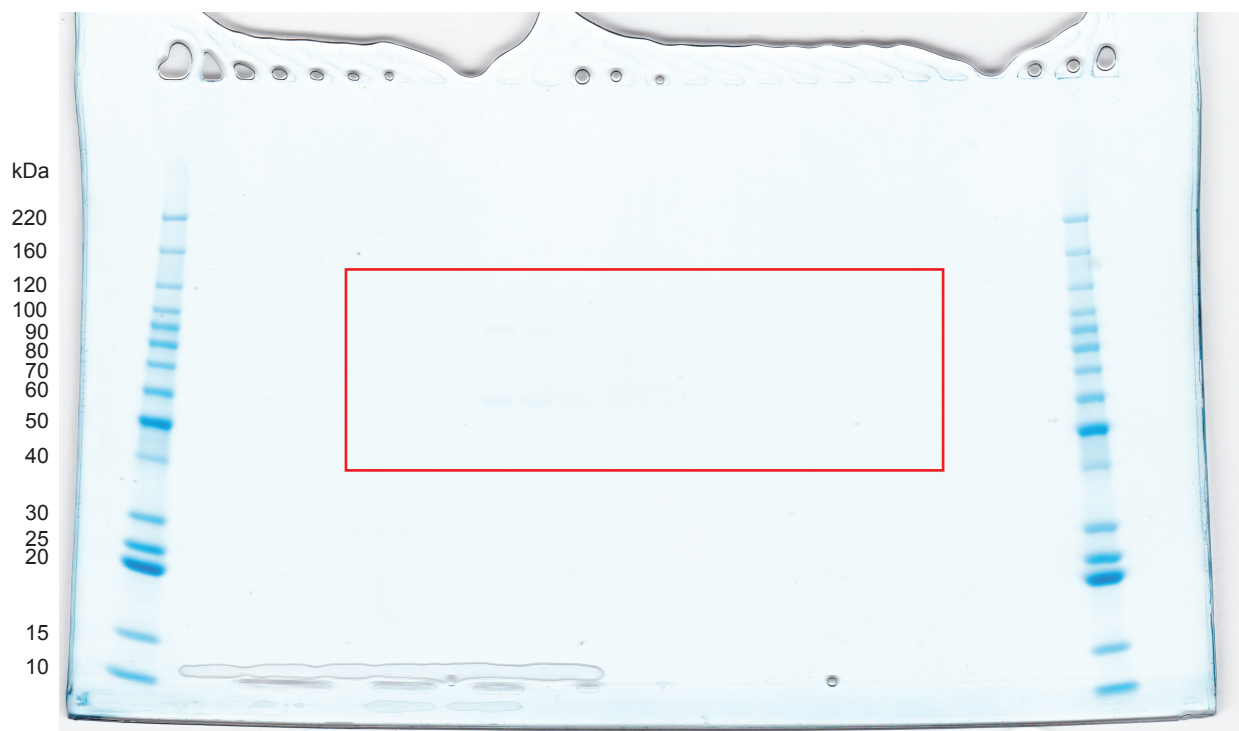

Figure 7B, DDK

Supplement: Figure 7—source data 6. [file elife-58571-fig7-data6.pdf]

## Figure 7 - source data 7

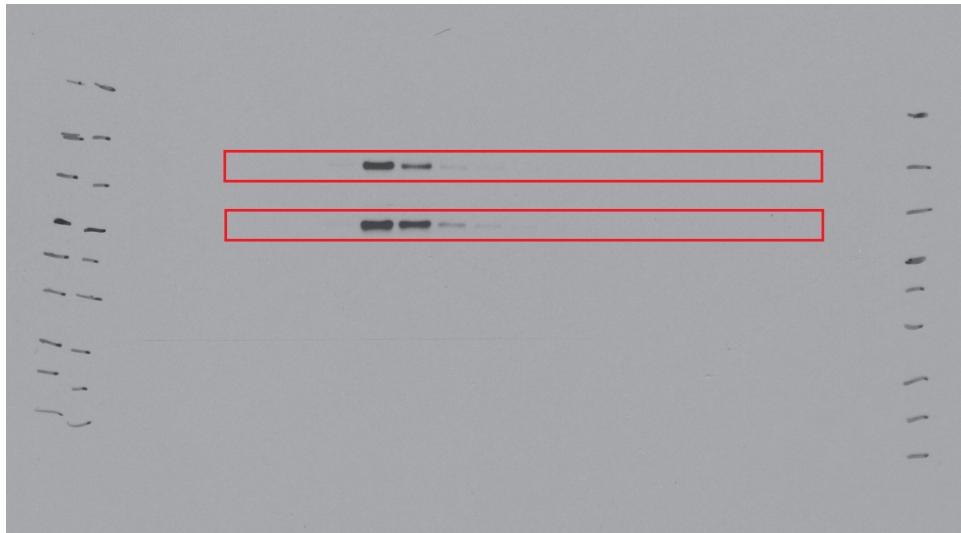

Figure 7B, DDK  
Immunoblot: Dbf4, Cdc7

Supplement: Figure 7—source data 7. [file elife-58571-fig7-data7.pdf]

Figure 7 - source data 8

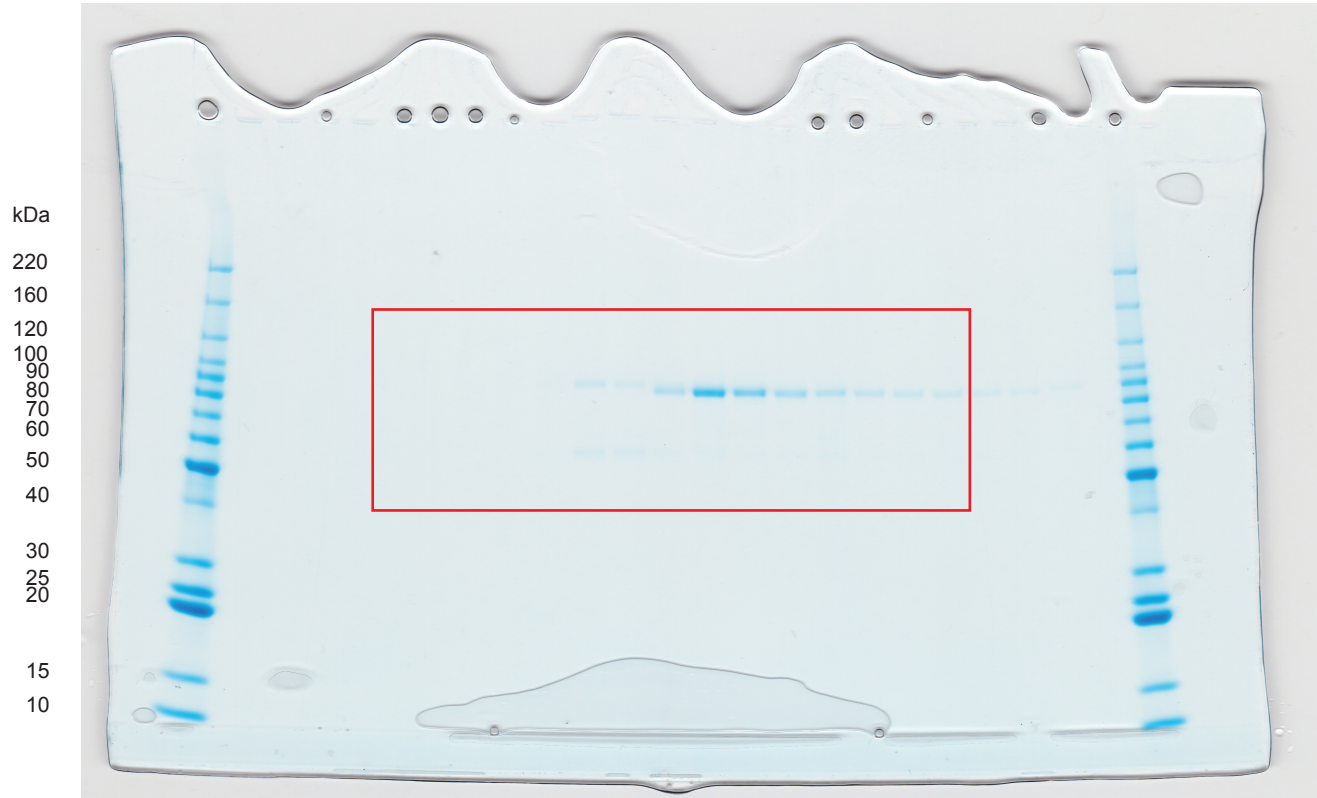

Figure 7B, Rad53-kd + DDK

Supplement: Figure 7—source data 8. [file elife-58571-fig7-data8.pdf]

Figure 7 - source data 9

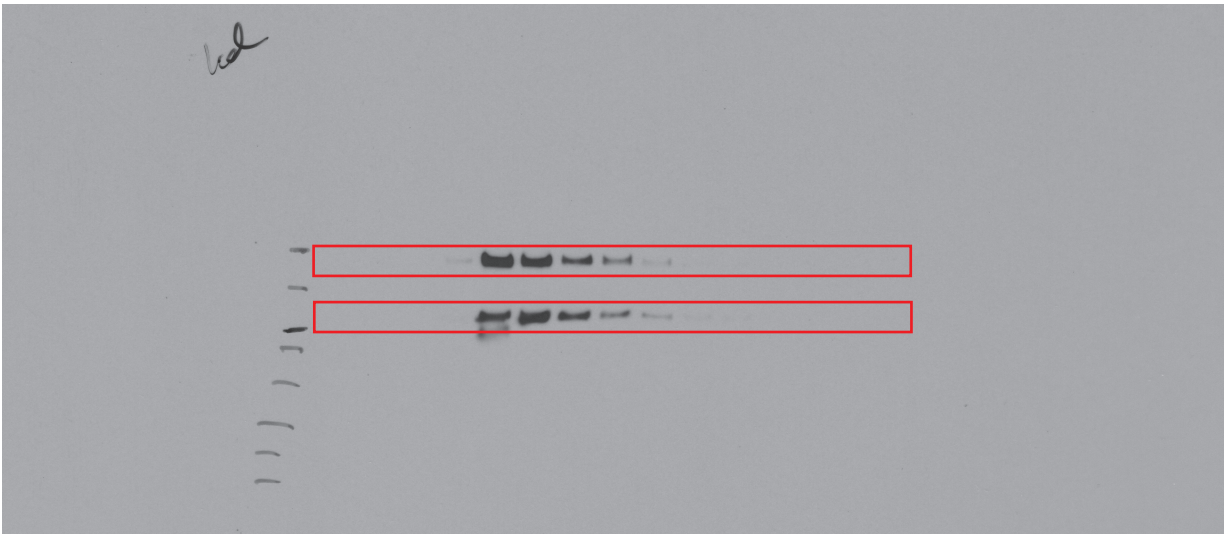

Figure 7B, Rad53-kd + DDK  
Immunoblot: Dbf4, Cdc7

Supplement: Figure 7—source data 9. [file elife-58571-fig7-data9.pdf]
